# Foreground Estimation in Neuronal Images With a Sparse-Smooth Model for Robust Quantification

**DOI:** 10.3389/fnana.2021.716718

**Published:** 2021-10-26

**Authors:** Shijie Liu, Qing Huang, Tingwei Quan, Shaoqun Zeng, Hongwei Li

**Affiliations:** ^1^Britton Chance Center for Biomedical Photonics, Wuhan National Laboratory for Optoelectronics, Huazhong University of Science and Technology, Wuhan, China; ^2^MoE Key Laboratory for Biomedical Photonics, Collaborative Innovation Center for Biomedical Engineering, School of Engineering Sciences, Huazhong University of Science and Technology, Wuhan, China; ^3^School of Computer Science and Engineering/Artificial Intelligence, Hubei Key Laboratory of Intelligent Robot, Wuhan Institute of Technology, Wuhan, China; ^4^School of Mathematics and Physics, China University of Geosciences, Wuhan, China

**Keywords:** neuronal images, foreground estimation, sparse-smooth model, robust quantification, enhancement

## Abstract

3D volume imaging has been regarded as a basic tool to explore the organization and function of the neuronal system. Foreground estimation from neuronal image is essential in the quantification and analysis of neuronal image such as soma counting, neurite tracing and neuron reconstruction. However, the complexity of neuronal structure itself and differences in the imaging procedure, including different optical systems and biological labeling methods, result in various and complex neuronal images, which greatly challenge foreground estimation from neuronal image. In this study, we propose a robust sparse-smooth model (RSSM) to separate the foreground and the background of neuronal image. The model combines the different smoothness levels of the foreground and the background, and the sparsity of the foreground. These prior constraints together contribute to the robustness of foreground estimation from a variety of neuronal images. We demonstrate the proposed RSSM method could promote some best available tools to trace neurites or locate somas from neuronal images with their default parameters, and the quantified results are similar or superior to the results that generated from the original images. The proposed method is proved to be robust in the foreground estimation from different neuronal images, and helps to improve the usability of current quantitative tools on various neuronal images with several applications.

## Introduction

Advanced development of 3D volume imaging techniques have enabled the generation of large-scale neuronal image at micron (Ragan et al., 2012; Chung et al., 2013; Chung and Deisseroth, 2013; Cai et al., 2019) and even submicron resolution (Li et al., 2010; Silvestri et al., 2012; Gong et al., 2013; Osten and Margrie, 2013; Economo et al., 2016; Winnubst et al., 2019), which facilitate the observation of complete neuron morphology of individual neuron in whole mammalian brain at molecular resolution. These techniques have provided huge and valuable datasets, and promote many fine studies in neuroscience research, including cell type identification, long range projection neuron reconstruction, neural circuit mapping, and neural modeling (Helmstaedter and Mitra, 2012; Osten and Margrie, 2013; Peng et al., 2015; Zeng and Sanes, 2017). Due to the complexity of neuronal structure in large-scale, the differences in sample preparation and imaging procedure, the neuronal images become huge, diverse and complicated. A large number of software tools (Rodriguez et al., 2009; Peng et al., 2010; Wang et al., 2011; Quan et al., 2013; Gala et al., 2014; Feng et al., 2015; Fürth et al., 2018) have been developed to quantify these challenging datasets, including soma localization (Quan et al., 2013; Yan et al., 2013; Frasconi et al., 2014; Ozcan et al., 2015; Kayasandik and Labate, 2016; He et al., 2018) and neurite tracing (Peng et al., 2010, 2011; Wang et al., 2011; Quan et al., 2013; Li R. et al., 2019; Winnubst et al., 2019; Zhou et al., 2020). However, most of these tools behave well on some specific datasets and experience the difficulties to deal with various neuronal images (Meijering et al., 2016; Fürth et al., 2018). Their performances decline in some cases, and users have to tune and balance multiple parameters carefully to obtain good quantified result. The main reason behind the situation is the lack of robust foreground estimation from neuronal images.

Foreground estimation from neuronal image is essential in soma localization and neurite tracing. Current soma segmentation methods and neurite tracing methods normally locate the initial positions of soma and neurites by finding the positions of local maximum intensity in the estimated foreground. Therefore, many algorithms (Lobregt et al., 1980; Yuan et al., 2009; Peng et al., 2011) need to set a series of thresholds for foreground estimation. However, the foreground intensities are usually inhomogeneous, and vary greatly across different image stacks or sub-stacks of an image. In some cases, the foreground intensities are lower than that of the background (Li S. et al., 2019). It is challenging to apply these methods on various neuronal image stacks or large neuronal datasets for the difficulty of finding proper threshold for every different image stack or sub-stack.

Machine-learning based methods are proposed to improve the foreground estimation from uneven neuronal images (Frasconi et al., 2014; Chen et al., 2015; Li R. et al., 2017; Mazzamuto et al., 2018; Li and Shen, 2020). Support vector machine (SVM) based methods use careful designed hand-crafted features to identify foreground signals whose features are consistent with the training set (Chen et al., 2015; Li S. et al., 2019), and improve the accuracy in inhomogeneous neurite tracing. Considering the summarized features are limited, the training set cannot cover the diversities of neuronal images. These methods are also computational complex and time-consuming, and they may need to construct corresponding training set for every sub-stack in a large-scale dataset. Deep-learning based methods (Frasconi et al., 2014; Li R. et al., 2017; Mazzamuto et al., 2018; Li and Shen, 2020; Huang et al., 2021) have been employed for cell identification and neuron reconstruction. These methods use deep convolutional network to extract deeper and more abundant features of neuronal images, and some also take advantage of traditional methods, such as mean-shift, Isomap algorithm (Frasconi et al., 2014), probabilistic blob detection (Mazzamuto et al., 2018), and content-aware adaptive voxel scooping (Huang et al., 2021), together to boost the accuracy in the foreground estimation from various neuronal images and even large-scale datasets significantly. While these methods need large-amount of training samples with manual annotations, which limits it usage in a biological laboratory and damages its generalization (Huang et al., 2020).

In this study, we propose a robust sparse-smooth model (RSSM) for foreground estimation from neuronal images. The proposed method is built based on two prior constraint: (1) Both the foreground and background are smooth, and the background is smoother. (2) The foreground is sparse as the foreground signals generally occupy a small rate compared to the whole volume. We combine these two prior constraint and build a convex optimization model to estimate the foreground. We evaluate RSSM on the neuronal images collected by light-sheet microscopy (Zhang et al., 2017; Yang et al., 2018) and fluorescence micro-optical sectioning tomography (fMOST) (Gong et al., 2013). The results suggested that RSSM estimated the foreground in different experimental conditions accurately, and was robust to various kinds of datasets with default parameters. We demonstrated that RSSM promoted some best-available tools to trace neurites successfully. Thus, RSSM boosted the robustness of these quantitative tools in soma location and neurite tracing from neuronal images.

## Materials and Methods

### Robust Sparse-Smooth Model (RSSM)

An optical neuronal image is normally composed of the foreground, the background and noise, which is given by:


(1) $$Y=I_{s}+B+I_{noise}$$

Where *Y* is the observed image. *I*_*s*_, *B*, and *I*_*noise*_ represent the foreground, background and noise image, respectively. The foreground is estimated based on two constraints: *I_s_* is sparse as the foreground signals generally occupy a small proportion in the volume; and the background is smoother than the foreground. These constraints are considered into the convex optimization problem for foreground estimation:


(2) $$\min\frac{1}{2}{||Y-I_{s}-B||}_{2}^{2}+\lambda_{1}{||I_{s}||}_{1}+\frac{1}{2}\lambda_{2}{||\nabla^{\left(k_{0}\right)}I_{s}||}_{2}^{2}+\frac{1}{2}\lambda_{3}{||\nabla^{\left(k_{1}\right)}B||}_{2}^{2}$$

Where $|||{|}_{2}^{2}$ is the square of L2 norm. || ||_1_ is L1 norm. λ_1_, λ_2_, and λ_3_ are weighting parameters. ${||\nabla^{\left(k_{0}\right)}I_{\text{s}}||}_{2}^{2}$ is given by:


(3) $$\begin{array}{c} {||\nabla^{\left(k_{0}\right)}I_{s}||}_{2}^{2}=\Sigma_{\left(x,y\right)}{||\nabla^{\left(k_{0}\right)}I_{s}\left(x,y\right)||}_{2}^{2}\\ ={||\nabla_{x}^{\left(k_{0}\right)}I_{s}\left(x,y\right)||}_{2}^{2}+{||\nabla_{y}^{\left(k_{0}\right)}I_{s}\left(x,y\right)||}_{2}^{2}\\ ={\left(k_{0}I_{s}\left(x,y\right)-\Sigma_{i=1}^{k_{0}}I_{s}\left(x-i,y\right)\right)}^{2}+\\ {\left(k_{0}I_{s}\left(x,y\right)-\Sigma_{i=1}^{k_{0}}I_{s}\left(x,y-i\right)\right)}^{2} \end{array}$$

Where (*x*, *y*) is the coordinate of a pixel in *I*_*s*_. *k*_0_ and *k*_1_ are smooth parameters. ${||\nabla^{\left(k_{1}\right)}B||}_{2}^{2}$ has the same definition as ${||\nabla^{\left(k_{0}\right)}I_{\text{s}}||}_{2}^{2}$. The constraint *k*_0_ < *k*_1_ indicates that the background is smoother than the foreground. In (2), the sparse term (second term) and the smooth term (third term) are combined to describe the sparsity and smoothness of the foreground. The smooth term estimates the foreground roughly without the sparse term. The noise couldn’t be removed well without the smooth term. The cooperative operation of the sparse term and the smooth term enhances the accuracy and robustness of the foreground estimation.

### Algorithms

We use proximal gradient descent (PGD) method (Xiao and Zhang, 2013) to solve (2). For simplification, we modify (2) as follows:


(4) $$\min F\left(I_{s},B\right)+\lambda_{1}{||I_{s}||}_{L1}$$

Where *F*(*I*_*s*_, *B*) is the sum of the first, third and forth terms in (2). According to PGD, the iterative formulas are given by:


(5) $$I_{s}^{k+1}=T\left(\max\left(I_{s}^{k}-\frac{1}{L_{s}}\frac{\partial F\left(I_{s},B\right)}{\partial I_{s}},0\right),3\right)$$

(6) $$B^{k+1}=\max\left(B^{k}-\frac{1}{L_{B}}\frac{\partial F\left(I_{s},B\right)}{\partial B},0\right)$$

Where *T*(*V*, 3) (Beck and Teboulle, 2009) is a function that process matrix *V*, and set to 0 if its element value is less than 3. We iteratively update the foreground *I*_*s*_ and the background *B* according to (5) and (6) until convergence. The final results are used as the estimated foreground and background.

Here, we briefly describe how to calculate the image gradient descent. From (2) and (3), we have


(7) $$\frac{\partial {||\nabla^{\left(k_{0}\right)}I_{s}||}_{2}^{2}}{\partial I_{s}}=\frac{\partial \left({||\nabla_{x}^{\left(k_{0}\right)}I_{s}||}_{2}^{2}+{||\nabla_{y}^{\left(k_{0}\right)}I_{s}||}_{2}^{2}\right)}{\partial I_{s}}=2{\left(\nabla_{x}^{\left(k_{0}\right)}\right)}^{T}\nabla_{x}^{\left(k_{0}\right)}I_{s}+2{\left(\nabla_{y}^{\left(k_{0}\right)}\right)}^{T}\nabla_{y}^{\left(k_{0}\right)}I_{s}$$

And thus,


(8) $$\frac{\partial F\left(I_{s},B\right)}{\partial I_{s}}=I_{s}+B-Y+\lambda_{2}{\left(\nabla_{x}^{\left(k_{0}\right)}\right)}^{T}\cdot \nabla_{x}^{\left(k_{0}\right)}I_{s}+\lambda_{2}{\left(\nabla_{y}^{\left(k_{0}\right)}\right)}^{T}\nabla_{y}^{\left(k_{0}\right)}I_{s}$$

(9) $$\frac{\partial F\left(I_{s},B\right)}{\partial B}=I_{s}+B-Y+\lambda_{3}{\left(\nabla_{x}^{\left(k_{1}\right)}\right)}^{T}\cdot \nabla_{x}^{\left(k_{1}\right)}B+\lambda_{3}{\left(\nabla_{y}^{\left(k_{1}\right)}\right)}^{T}\nabla_{y}^{\left(k_{1}\right)}B$$

The calculation of (7) is key to the gradient descent calculation in (8) and (9). Detailed steps are described as follows:

**Step 1**. Input image *I*_*s*_ with *m* rows and *n* columns; Set a template vector denoted by *tem* with the length *k*_0_+1; the first *k*_0_ elements is set to −1 and the last one is *k*_0_.
**Step 2**. Calculate $Is^{\nabla_{x}}=\nabla_{x}^{\left(k_{0}\right)}I_{s}$ : Assign a zero matrix with *m*+*k*_0_ rows and *n* columns into $I_{s}^{\nabla_{x}}$, and update the element values of *Is*^∇*x*^ ranging from (*k*_0_+1) *^*th*^* row to *m*^*th*^ row as follows.

(10) $$I_{s}^{\nabla_{x}}\left(i+k_{0},j\right)=\sum_{l=1}^{l=k_{0}+1}I_{s}\left(i+l-1,j\right)tem\left(l\right)\;\left(i=1,2,\cdots ,m-k_{0};j=1,2,\cdots ,n\right)$$

**Step 3**. Calculate ${\left(\nabla_{x}^{\left(k_{0}\right)}\right)}^{T}\nabla_{x}^{\left(k_{0}\right)}I_{s}$ :

(11) $${\left(\nabla_{x}\right)}^{T}\left(I_{s}^{\nabla_{x}}\left(i,j\right)\right)=\sum_{l=1}^{l=k_{0}+1}I_{s}^{\nabla_{x}}\left(i+l-1,j\right)tem\left(k_{0}+1-l\right)\;\left(i=1,2,\cdots ,m;j=1,2,\cdots ,n\right)$$

${\left(\nabla_{y}^{\left(k_{0}\right)}\right)}^{T}\nabla_{y}^{\left(k_{0}\right)}I_{s}$ and the gradient image of B are also calculated using the similar above procedure.

Then, the gradient descent steps 1/L_*s*_ and 1/L_*B*_ are need to be fixed. According to Cauchy-Lipschitz Theorem, the convenient results are obtained if the following equations are satisfied:


(12) $$L_{s}\geq \lambda_{\max}\left(E+\lambda_{2}{\left(\nabla_{x}^{\left(k_{0}\right)}\right)}^{T}\nabla_{x}^{\left(k_{0}\right)}+\lambda_{2}{\left(\nabla_{y}^{\left(k_{0}\right)}\right)}^{T}\nabla_{y}^{\left(k_{0}\right)}\right)$$

(13) $$L_{\text{B}}\geq \lambda_{\max}\left(E+\lambda_{3}{\left(\nabla_{x}^{\left(k_{1}\right)}\right)}^{T}\nabla_{x}^{\left(k_{1}\right)}+\lambda_{3}{\left(\nabla_{y}^{\left(k_{1}\right)}\right)}^{T}\nabla_{y}^{\left(k_{1}\right)}\right)$$

Where λ_*max*_ (.) represents the largest eigenvalue of the matrix induced by operation on images; *E* is the unit matrix; ${\left(\nabla_{x}^{\left(k_{0}\right)}\right)}^{T}\nabla_{x}^{\left(k_{0}\right)}$ performs two convolution operations on an image (Steps 2 and 3), and induces a matrix. The largest eigenvalue of the induced matrix is less than (*k*_0_ × *k*_0_ + *k*_0_)^2^ (Milinazzo et al., 1987). So, we have


(14) $$L_{s}\geq 1+2\lambda_{2}{\left(k_{0}\times k_{0}+k_{0}\right)}^{2}$$

(15) $$L_{B}\geq 1+2\lambda_{3}{\left(k_{1}\times k_{1}+k_{1}\right)}^{2}$$

Parameter λ_1_ is the weighting parameter of the foreground sparse term, and set to a fixed value of 0.1. As the background is usually smoother than that of the foreground, *k*_0_ should be smaller than *k*_1_. *k*_0_ is set to 2 and *k*_1_ is 5. The smooth term calculates the sum of the gradient values of the corresponding foreground and background. For neuronal images, the smooth term of the background is normally smaller than the smooth term of the foreground, and the ratio of the two smooth terms fluctuates between a certain range. To satisfy Eqs. 14 and 15 and make the two terms work well for neuronal image, λ_2_ should be smaller than λ_3_, we set λ_2_ to 0.1 and λ_3_ to 0.5 based on experience and experiment. In all of our experiments, these parameters are kept unchanged.

The initial iterative image of *I*_*s*_ and *B* are determined by our previous works (Quan et al., 2013). We roughly provide a threshold value (the median value of all pixel values in the observed image), let pixel value of the observed image less than this threshold, and then convolute it with a Gaussian kernel (convolution number: 20). The convoluted image and the difference of the observed image and the convoluted image are regarded as the initial estimated background and foreground, respectively.

### Evaluation of Quantitative Methods

We quantified the estimated foreground and its corresponding original images with some available tools (Wang et al., 2011; Quan et al., 2013; Feng et al., 2015) on soma localization and neurite tracing. The metrics of precision, recall and F1 score are used for quantitative evaluation (Brown et al., 2011; Quan et al., 2016; Li S. et al., 2019). Precision is defined as the ratio of true positive (TP) number to all the searched object numbers via the automatic methods. Recall is defined as the ratio of TP number to all the object numbers searched by manual segmentation. F1 score is the harmonic mean of the recall and precision. In soma locating evaluation, the true positive position of the located somas via automatic methods are manually checked. In neurite tracing evaluation, for any skeleton point obtained via automatic methods, if the closest distance between the point and the gold standard is less than 6 μm, it is regarded as a TP point (Quan et al., 2016; Li S. et al., 2019). In the paper, the manual segmented result of soma or traced skeleton points are used as the gold standard.

### Experimental Setup

The proposed RSSM method was implemented using C++ language and packaged into a software named RSSM, which can be accessed via https://github.com/LGBluesky/RSSM/releases. All of the experiments were performed on a personal computer with Intel(R) i7-6850K CPU, 3.60 GHz, 64 GB RAM, and NVIDIA 1080Ti. In the computation, we transformed the iterative calculation to convolution operation and also used cuda to accelerate the foreground estimation.

## Results

**FIGURE 1:**
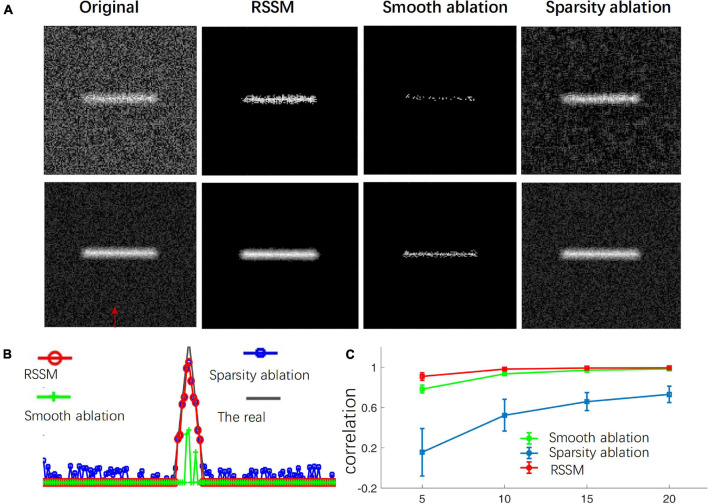
Ablation study of the proposed RSSM method to validate the role of the sparse and smooth terms in foreground estimation. **(A)** Shows simulated neurite image (first column), estimated foreground via RSSM (second term), the smooth ablation model (third term), and the sparsity ablation model (fourth term). **(B)** Shows the image intensity distribution of the original image and the estimated foreground via three models along the direction that pointed out by a red arrow in **(A)**. The real means the image that only contains the foreground. **(C)** Shows the correlation between the image intensity distribution curves in **(B)**. The *x*-axis represents different groups of images and each group has the same foreground intensity.

We performed ablation study of the proposed RSSM method to validate the importance of the sparse term and the smooth term in the foreground description and estimation of neuronal image. We compared the performances of the RSSM method, a sparsity ablation model and a smooth ablation model on some simulated images with image size 128 × 128. We deleted the smooth term and the sparse term in Eq. 2 and regarded as the smooth ablation model and sparsity ablation model, respectively. Other configurations remained the same. To generate the simulated images, we first used a thin rectangle as the foreground, and the background value was set to 200. Then, the images were convoluted by a mean filter of size 7 × 7. We finally added uniform random noises ranging from [−2.5, 2.5] to the images. Three groups of images (each group 50 images) with foreground intensities that were 5, 10, 15, and 20 higher than that of the background were generated. Figure 1 shows the foreground estimation performances of the three models on the simulated images, whose foreground intensities were only 5 and 15 higher than that of the background. As we can see, the smooth ablation model effectively suppressed background noises, while it lost most of the foreground signals in both cases. It failed to discern the foreground and the background when their intensity differences were small (Figure 1A). The sparsity ablation model could keep the foreground signals, while it was hard to remove all the noises. The proposed RSSM method, which combined the advantages of the two models, suppressed noisy background meanwhile almost kept all the foreground signals. We also calculated the image intensities in the original and estimated foreground images via three models to for further illustration. The intensities along the direction that labeled by a red arrow in Figure 1A were shown in Figure 1B. The correlation of the estimated foreground intensity distribution curves and that of the original image are shown in Figure 1C. The proposed RSSM model achieved similar foreground intensity distribution to that of the original image, and suppressed background noises interference. The correlation of RSSM were obviously higher than that of using the sparsity ablation model and the smooth ablation model alone. These results proved that the combination of the sparsity and the smooth terms in the optimization problem were effective to the accurate foreground estimation.

**FIGURE 2:**
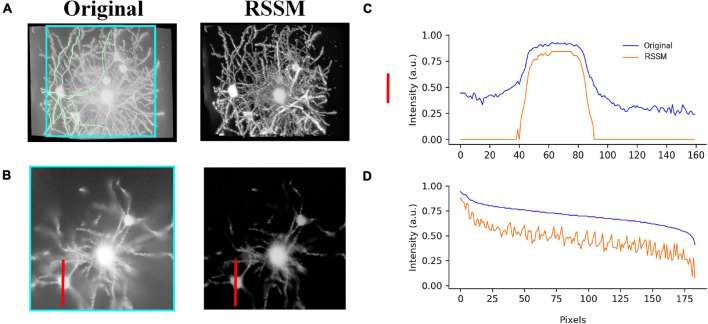
Foreground estimation via RSSM from 3D fMOST neuronal image. **(A)** A neuronal image (left) and its foreground estimation (right) in 3D view. **(B)** 2D view of a selected slice in **A** (marked by green square) and its corresponding foreground estimation. **(C,D)** Show the image intensity distribution of the neuronal image and the estimated foreground image along a red line in **(B)** and traced neurite skeleton points (green lines in **A**), respectively.

We also performed the proposed RSSM method on a 3D optical neuronal image to validate the effectiveness of RSSM on foreground estimation. The image stack contained multiple neurons and many nearby neurites. It was collected using fMOST system (Gong et al., 2013), its size was 428 × 500 × 287 and spatial resolution was 0.3μ*m* × 0.3μ*m* × 1μ*m*. Figures 2A,B show the 2D view of a selected slice and 3D view of the neuronal image and the estimated foreground image, respectively. In the neuronal image, the background was high and the foreground was fuzzy. Using RSSM, most of the background noises, including the haze noise, were removed and nearly all the neuron signals were kept. Figure 2C shows the intensity distribution of the two images along the red line in Figure 2A. In the estimated foreground image, the background intensities were almost close to 0, and the foreground intensities were close to the original image. As the noise inference was almost suppressed in the estimated foreground image, the complexity of the image was reduced. The image intensity distribution of some traced neurite skeleton points via NeuroGPS-Tree (Quan et al., 2016) (green curves in Figure 2A) of the original image and the estimated foreground image were shown in Figure 2D. It can be seen, the two curves had similar intensity distribution trends. As the estimated foreground image only contained the foreground signals and also some rounding errors caused during calculation, the intensity distribution curve of the estimated foreground image seemed rougher and sharper than that of the original image. Considering the background intensities were close to zeros, and the calculated foreground intensities were larger than 0, the proposed RSSM could simplify the foreground estimation compared to the original image.

**FIGURE 3:**
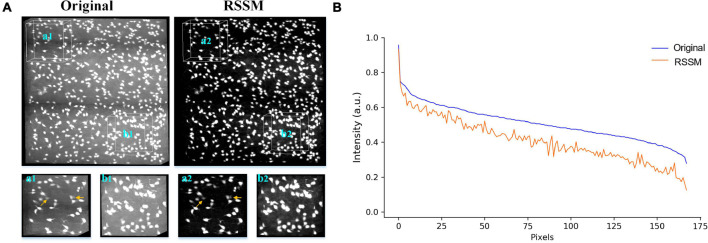
Foreground estimation via RSSM from 3D soma image. **(A)** Shows a 3D soma image collected using light-sheet microscopy and its corresponding estimated foreground image. Two sub-stacks that contained sparsely and densely distributed somas and inhomogeneous background were showed in the bottom. Somas with weak intensities in a1-a2 are pointed by arrows. **(B)** Shows the image intensity distribution of all the manual located somas of the original image and the estimated foreground image.

We performed the proposed RSSM method on a soma image stack to validate the effectiveness of RSSM for soma estimation. The image stack was collected using collected with light-sheet microscopy (Yang et al., 2018), its size was 600 × 600 × 200 and its spatial resolution was 1μ*m* × 1μ*m* × 1μ*m*. As seen in Figure 3A, the intensity distributions of the soma and the background were large, and intensities changed obviously across different sub-stacks. We exhibited the original image and the estimated foreground image of two sub-stacks that contained sparsely and densely distributed somas (Figure 3A). The proposed RSSM method suppressed the inhomogeneous background noise with different intensity distribution range, and estimated the somas with weak intensities from noisy background (pointed out by arrows in Figures 3a1,a2) successfully. We manually located the positions of all somas using NeuroGPS-Tree software, and calculated their image intensity distributions of the original image and the estimated foreground image (see Figure 3B). These qualitative and quantitative results suggested that RSSM could estimate the foreground of somas from inhomogeneous background successfully.

**FIGURE 4:**
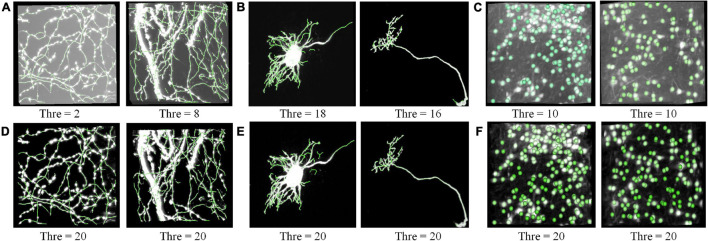
RSSM facilitates robust quantification on the estimated foreground. **(A)** Neurites images collected using fMOST system and their corresponding tracing results. **(B)** Neuronal images with a complete single neuron from the public BigNeuron (left) and Diadem (right) dataset and their tracing results. **(C)** Neuronal images collected using light-sheet microscopy and their corresponding soma localization results. **(D–F)** Are the estimated foreground images by RSSM and their corresponding quantitative results. The NeuroGPS-Tree software is used for neurite tracing and soma localization. *Thre* is a key threshold parameter in NeuroGPS-Tree for neurite tracing and soma localization.

We performed the proposed RSSM method on various neuronal images to validate it could facilitate robust quantification. Six varied optical neuronal images, which contained different foreground contents (somas, neurites, and complete neurons) and acquired using different imaging systems, were selected for this validation and divided into three groups. The first group contained neuronal images that collected using fMOST system (Gong et al., 2013) and had axon signals (Figure 4A). The second group contained neuronal images that from the public BigNeuron (Peng et al., 2015) and Diadem (Brown et al., 2011) datasets, and had complete neuronal structures including somas, dendrites and axons (Figure 4B). The third group contained neuronal images that collected using light-sheet microscopy (Yang et al., 2018) and had soma signals (Figure 4C). We applied a quantitative tool NeuroGPS-Tree on the original images and their corresponding estimated foreground images of the three groups. The NeuroGPS-Tree obtains the initial foreground segmentation using local threshold with a key threshold parameter (defined as *Thre*). Parameter *Thre* is important to the quantitative analysis, as it determines the initial soma positions in the soma localization, the initial seed points and tracing termination conditions in neurite tracing. To obtain good quantification results from the original images with varied background, we tuned this parameter carefully for every different original image and kept other parameters in default. For the estimated foreground image by RSSM, *Thre* was set to a fixed value 20 and other parameters were in default. Figure 4 shows the almost best available results of the original image with *Thre* ranged from 2 to 18, and the results of the estimated foreground with fixed *Thre* value. The quantitative evaluation results on the original and estimated foreground images can be seen in Table 1 (data 1–6 correspond to the six images in Figure 4 from left to right in sequence). The F1 scores on the original image using manual tuned parameter were between 0.84 and 0.95, with average value 0.90 for neurite tracing, and between 0.70 and 0.89 with average value 0.79 for soma localization. The F1 scores on the estimated image using fixed parameter were between 0.82 and 0.95 with average value 0.88 for neurite tracing, and between 0.82 and 0.84 with average value 0.83 for soma localization. The processing time of RSSM on these images were between 0.3 and 17.5 s. The quantitative results of the estimated foreground achieved similar results of the best available results on the original images. These performances prove that the proposed RSSM method provides a way for robust quantification of the quantitative tool of diverse neuronal image without adjusting parameters.

**TABLE 1:** Evaluation of NeuroGPS-Tree on neuronal images for neurite tracing and soma localization.

**Data id**	**Volume**	**Size [MB]**	**F1 scores**		**Precision**		**Recall**		**Time [s]**
			**Original**	**RSSM**	**Original**	**RSSM**	**Original**	**RSSM**	
1	301 × 301 × 86	7.61	0.95	0.93	0.98	0.90	0.93	0.97	1.3
2	301 × 301 × 172	14.89	0.84	0.84	0.79	0.95	0.90	0.75	2.6
3	1,024 × 1,024 × 113	113.01	0.86	0.82	0.94	0.75	0.80	0.85	17.5
4	512 × 512 × 67	16.71	0.94	0.95	0.92	0.96	0.97	0.93	2.6
5	120 × 120 × 120	1.67	0.70	0.84	0.81	0.85	0.62	0.83	0.4
6	120 × 120 × 120	1.67	0.89	0.82	0.93	0.77	0.85	0.88	0.3

**FIGURE 5:**
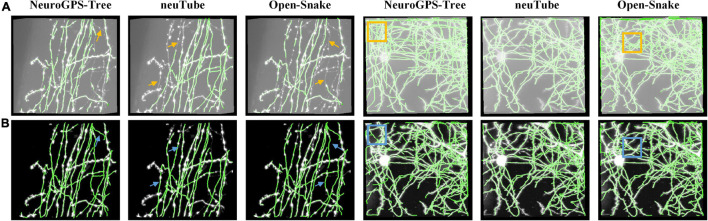
RSSM facilitates the usage of some typical quantitative software tools. **(A)** Two fMOST neuronal images and their corresponding tracing results by software NeuroGPS-Tree, neuTube, and Open-Snake with selected parameters. **(B)** The estimated foreground images by RSSM and their corresponding tracing results by these tools with default parameters.

To validate RSSM could facilitate and simplify the usage of some typical quantitative software tools, including NeuroGPS-Tree, neuTube, and Open-Snake (Rodriguez et al., 2009; Quan et al., 2013; Feng et al., 2015), we compared the neurite tracing results of these tools on original images and the estimated foreground images. We selected two fMOST neuronal images for testing. One had sparsely distributed neurites, and its size was 301 × 301 × 138. The other had densely distributed neurites, and its size was 428 × 500 × 148. Figure 5 shows the comparative results of these tools on the original images and the estimated foreground images. For original images, we selected proper parameters for these tools to obtain good results. For the estimated foreground images, we used default parameters for neurite tracing. Table 2 showed the quantitative evaluation results of these tools on the two kinds of images. The processing time of RSSM on these images were between 2.1 and 4.9 s. The F1 score of these tools on the original images with the selected parameters were between 0.73 and 0.94, and the average scores of NeuroGPS-Tree, neuTube and Open-Snake were 0.86, 0.87, and 0.76, respectively. The F1 score of these tools on the estimated foreground images with default parameters were between 0.89 and 0.98, and the average scores of NeuroGPS-Tree, neuTube and Open-Snake were 0.94, 0.90, and 0.94, respectively. These results indicate that the proposed RSSM method boosts the availability of these typical quantitative tools.

**TABLE 2:** Evaluations of NeuroGPS-Tree (NTree), neutube, Open-Snake (Snake) for neurite tracing.

**Data id**	**Volume**	**Size [MB]**	**Time [s]**		**Original**			**RSSM**	
				**NTree**	**neuTube**	**Snake**	**NTree**	**neuTube**	**Snake**
1	301 × 301 × 138	11.95	2.1	0.78	0.90	0.78	0.89	0.90	0.92
2	428 × 500 × 148	30.22	4.9	0.94	0.84	0.73	0.98	0.89	0.95
Average	–	21.09	3.5	0.86	0.87	0.76	0.94	0.90	0.94

**FIGURE 6:**
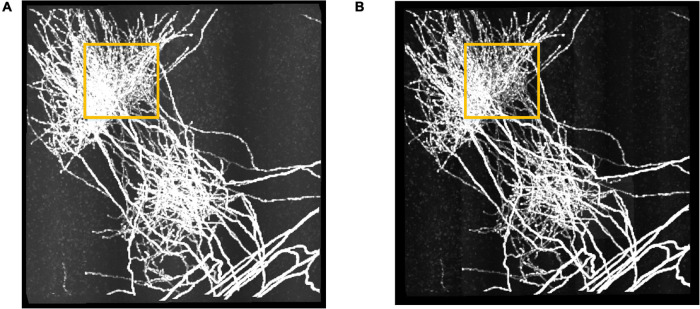
The performance of RSSM on a large neuronal dataset. **(A)** Shows a fMOST neuronal dataset with size 2,560 × 2,560 × 512 (∼6.4 GB). **(B)** Shows the corresponding estimated foreground by RSSM. Some haze noises in the marked region (yellow) was suppressed by RSSM, and the visualization of the estimated foreground was clearer.

We further performed the proposed RSSM method on a large fMOST neuronal dataset to validate that RSSM could be applied to large-scale images. The dataset was about 6.4 gigabyte (GB), and its size was 2,560 × 2,560 × 512. Limited by the hardware configuration, the maximum image size that can be processed was about 512 × 512 × 512. To process the large dataset, we divided it into 25 sub-stacks of size 512 × 512 × 512, applied RSSM on these sub-stacks, and then stitched the processed sub-stacks in sequence to obtain the final result. Figure 6 shows the performance of RSSM on the large dataset with inhomogeneous foreground and background intensities. RSSM effectively suppressed the scatter noises and haze noises in every different sub-stack, and kept almost all the neurite signals, even for the weak neurites. As marked out by the yellow rectangles, the neurite visualization of estimated foreground image was clearer than the original image after eliminating most of the noises. The average processing time for a sub-stack of size 512 × 512 × 512 was about 20 s, and the total processing time for the large neuronal dataset was 506 s with average processing speed 0.76 GB/minute. The performance on the large neuronal dataset indicates that the proposed RSSM is a general foreground estimation method, and can be applied on large-scale neuronal images with default parameters.

## Discussion

In this study, we proposed a robust smooth-sparse model (RSSM) for foreground estimation from various neuronal images. The proposed method combined the sparsity term and the smoothness term of the foreground and background for foreground estimation, and accurately estimated the foreground signals from varied background. RSSM reduces the complexity of the neuronal image by suppressing the high noises, and nearly keeps all the foreground information as the original images do in the neurite tracing or soma localization. Therefore, the robust quantifications can be achieved from the estimated foreground images.

Quantization of the neuronal image is regarded as the bridge from imaging datasets to knowledge discovery. Many tools (Peng et al., 2010; Wang et al., 2011; Quan et al., 2013; Feng et al., 2015; Zhou et al., 2020) have been proposed for this purpose. Due to the complexity and diversity of neuronal images, most of the quantitative tools behave well on some specific datasets. To the best of our knowledge, there is no tool that keeps competitive in all cases. In general, these quantitative methods have their own merits and deficiencies, which hinders the robust quantitative results. They usually need complex parameters adjustment to obtain good performances and avoid failure in some cases, which is time-consuming and experience dependency. The proposed RSSM method obtains the consistent results by estimating the inhomogeneous foreground and suppressing various backgrounds, which reduces the complexity and diversity of neuronal images. Thus, the estimated foreground can be easily quantified with some tools including NeuroGPS-Tree (Quan et al., 2016), neuTube (Feng et al., 2015), and NeuroStudio (Rodriguez et al., 2009). The proposed RSSM method is proved to be robust in the foreground estimation from various images that had different contents and collected using different imaging systems and methods (see Figures 2–6), and facilitate robust quantitative results of current tools.

The proposed RSSM method decomposes image into different items to build the model and estimates the foreground by solving optimization problem, which might look similar to the method of Robust Principal Component Analysis (RPCA) (Candès et al., 2011; Bouwmans et al., 2018). While there are great differences between the two methods. The model construction of the two methods were different. RPCA decomposes an image into the foreground and the background, and supposes one is low-rank and the other is sparse. This assumption is suitable to some cases such as video processing. While for neuronal images, the background is not low-rank, and the foreground of most neuronal images are usually irregular and high rank. The number, shape, position, intensity distribution and structure of neuronal signals varied greatly across different image stacks, which make the foreground image complex and unordered. The proposed RSSM method uses the prior sparsity and smooth constraints of the morphology of neuronal images to build the model. As seen in Figure 1, both the two terms contribute to the foreground estimation and facilitate robust results.

We noted that many machine learning-based methods are used for estimating foreground from neuronal images (Chen et al., 2015; Li S. et al., 2019). For machine learning based methods, the identification accuracy depends on the quality of their training datasets. When the features of the testing datasets are different from the training datasets, the predicted foreground may be far deviated from the reality. For neuronal images collected using different optical systems (Silvestri et al., 2012; Yang et al., 2018), robust estimation of various foregrounds by a machine learning-based model is difficult. Compared with machine learning based methods, the sparse-smooth model may not be competitive in some specific datasets, while it can provide the relative accurate and robust estimation of the foreground from various neuronal images collected using different systems. RSSM balanced the robustness and accuracy of quantifications, and can be used to simplify some following applications with default parameters.

The robust foreground estimation has lots of potential applications in quantifying neuronal images. Besides boosting the availability of current quantitative tools (Rodriguez et al., 2009; Peng et al., 2010; Quan et al., 2013; Feng et al., 2015), the estimated foregrounds could also be used to help some machine learning-based methods to construct their training datasets. Considering the diversity of the neuronal images, the training datasets of an image are usually obtained from the initial predicted results of the image itself for better prediction. This operation depends on the initial foreground identification, as these methods use the initial foreground to construct the positive samples and the others to construct the negative samples. Obviously, inaccurate foreground estimation would lead to the following inaccurate predictions. This case is unavoidable due to the lack of robust foreground estimation. The proposed RSSM method has the potential to solve the problem and help the machine learning methods to build better training samples. The performance and fast processing speed (0.76 GB/minute) of RSSM on the large neuronal dataset (Figure 6) also indicate that RSSM has the potential to be a general foreground estimation to promote large-scale neuronal image tracing and reconstruction. We will further optimize the engineering of our RSSM software to reduce its calculation and accelerate the processing speed for large images.

## Conclusion

We proposed a robust sparse-smooth model to estimate the foreground of neuronal images based on two prior constraints. We verified the effectiveness of the prior constraints by the ablation study. The proposed RSSM can perform robust foreground estimation, eliminate the background from various neuronal images, and reduce the complexity of neuron images. We further demonstrated that RSSM can boost the availability of typical quantitative tools to avoid complex parameters adjustment in quantization. RSSM also has the potential to be used in large-scale neuronal images and other tubular medical images such as vessel images.

## Data Availability Statement

The datasets presented in this study can be found in online repositories. The names of the repository/repositories and accession number(s) can be found below: https://pan.baidu.com/s/19KpeD9qCOXaDAa7hcHspLw with password r3cb.

## Author Contributions

HL and TQ conceived the project and corrected the manuscript. SL, QH, and TQ designed the algorithm. SL and QH wrote the manuscript. SZ produced the dataset. All authors contributed to the article and approved the submitted version.

## Conflict of Interest

The authors declare that the research was conducted in the absence of any commercial or financial relationships that could be construed as a potential conflict of interest.

## Publisher’s Note

All claims expressed in this article are solely those of the authors and do not necessarily represent those of their affiliated organizations, or those of the publisher, the editors and the reviewers. Any product that may be evaluated in this article, or claim that may be made by its manufacturer, is not guaranteed or endorsed by the publisher.
